# Diagnosis and treatment of latent tuberculosis in patients with multiple sclerosis, expert consensus. On behalf of the Colombian Association of Neurology, Committee of Multiple Sclerosis

**DOI:** 10.1177/2055217317752202

**Published:** 2018-01-17

**Authors:** Carlos Navas, Carlos A Torres-Duque, Joe Munoz-Ceron, Carlos Álvarez, Juan R García, Luis Zarco, Lázaro A Vélez, Carlos Awad, Carlos Alberto Castro

**Affiliations:** Department of Neurology, Clínica Universitaria Colombia, Bogotá, Colombia; Fundación Neumológica Colombiana, Colombia; Universidad de la Sabana, Bogotá, Colombia; Universidad del Rosario, Bogotá, Colombia; Clínica Universitaria Colombia – Hospital MEDERI Bogotá, Colombia; Department of infectology, Clinica Universitaria Colombia, Bogotá, Colombia; Departamento de Neurología, Clínica de Marly, Bogota Colombia; Department of Neurology, Hospital de San Ignacio, Universidad Javeriana, Bogotá, Colombia; Department of Internal Medicine, Faculty of Medicine, University of Antioquia, Medellin, Colombia; Deparmet of pulmonology, Hospital Santa Clara, Bogotá, Colombia; SIIES Research and Education in Health, Bogotá, Colombia

**Keywords:** Multiple sclerosis, tuberculosis, latent tuberculosis, consensus development, diagnosis, therapeutics

## Abstract

**Background:**

Multiple sclerosis is an inflammatory and neurodegenerative demyelinating disease. Current treatment of multiple sclerosis focuses on the use of immunomodulatory, immunosuppressant, and selective immunosuppressant agents. Some of these medications may result in high risk of opportunistic infections including tuberculosis.

**Objective:**

The purpose of this study was to obtain consensus from a panel of neurologists, pulmonologists, infectious disease specialists, and epidemiology experts regarding the diagnosis, treatment, and monitoring of latent tuberculosis in patients with multiple sclerosis.

**Methods:**

A panel of experts in multiple sclerosis and tuberculosis was established. The methodological process was performed in three phases: definition of questions, answer using Delphi methodology, and the discussion of questions not agreed.

**Results:**

Tuberculosis screening is suggested when multiple sclerosis drugs are prescribed. The recommended tests for latent tuberculosis are tuberculin and interferon gamma release test. When an anti-tuberculosis treatment is indicated, monitoring should be performed to determine liver enzyme values with consideration of age as well as comorbid conditions such as a history of alcoholism, age, obesity, concomitant hepatotoxic drugs, and history of liver disease.

**Conclusion:**

Latent tuberculosis should be considered in patients with multiple sclerosis who are going to be treated with immunomodulatory and immunosuppressant medications. Transaminase level monitoring is required on a periodic basis depending on clinical and laboratory characteristics. In addition to the liver impairment, other side effects should be considered when Isoniazid is prescribed.

## Introduction

Multiple sclerosis (MS) is a chronic inflammatory and neurodegenerative disease of the central nervous system (CNS) caused by lymphocytic infiltration, neuro-inflammation associated with CNS-resident cells, in particular microglia and macrophages and neurodegeneration, this condition is associated with high disability and a heavy burden of disease.^1^ MS is hypothesized to be multifactorial in origin, involving genetic, environmental, and immunological factors; the latter considered the main etiology leading to disability in young adults over many years. The main clinical manifestations depend on the localization of brain and/or spinal cord lesions that affect visual pathway, sensory and motor function, and cognitive skills. MS is classified according to it is clinical course as relapsing–remitting, primary progressive, and secondary progressive.^1,2^ The prevalence of MS per 100.000 inhabitants varies according to the geographical region and methodology, the reports in Latin America fluctuate between 0.83 to 38.2,^3^ in USA 110.7 to 192.1^4^, and Europe 15.78 to 252.95.^5^

Based on mechanisms of action and potential effects on the immune system, current treatments of MS are immunomodulatory agents, selective immunosuppressants or immunosuppressants. The first group consists of glatiramer acetate, interferon β-1b, and interferon β-1a in its SC and IM presentation. Fingolimod, natalizumab, alemtuzumab, rituximab, dimethyl fumarate, ocrelizumab, daclizumab, and teriflunomide are drugs with varying degrees of immunosuppressant properties. Taking into account this concept, MS-modifying drugs can bring about a risk of opportunistic infections including progression of a primary tuberculosis (TB) infection or reactivation of latent TB due to impact on cellular immunity.^6–8^

Approximately two billion people worldwide are infected with *Mycobacterium tuberculosis* (*M. tuberculosis*) and about one in 10 will develop TB at some point of their lives. Although TB mortality has decreased significantly, in 2014 the World Health Organization (WHO) estimated that there were 9.6 m new TB cases worldwide (3.2 m women and one million children), and 1.2 m deaths from TB. Twelve percent of TB cases are human immunodeficiency virus (HIV) co-infected and it is estimated that 5% had multi-drug resistant (MDR) TB (480.000 new cases of MDR TB in 2014). The TB global incidence is 133 per 100,000 population.^9,10^

In Colombia, there are 11,000 new reported cases of TB each year, with little variation over the last 10 years: in 2006, 11,122 cases of TB were reported with an incidence rate of 24 per 100,000 people^11^ and in 2014, 12,824 cases were reported with an incidence rate of 24.8 per 100,000 people.^10^ Although mortality has decreased significantly in Colombia, the global situation has not improved, probably due the impact of HIV co-infection and MDR TB.^12–14^

The prevalence of TB infection in addition to the impact of approved treatments on the immune system justifies the need of identifying risk groups to screen for latent TB in MS patients, to avoid active TB resulting from progression of a primary infection or activation of a latent TB.^8^ Currently, there is limited information with respect to parameters to address patients with MS and their probability of having latent TB. This gap in knowledge led a group of experts to meet in order to determine consensus-based guidelines on diagnosis, treatment, and follow-up of these patients.

Although MS is a condition that affects the CNS fundamentally, the immunological complications related to the treatment justify a comprehensive approach, including other specialists who are to a variable extent in charge of taking care of these patients.

According to the former arguments the main goal of this expert task force was to review the evidence and to suggest actions to study latent TB in individuals with MS.

## Materials and methods

**Figure 1.**
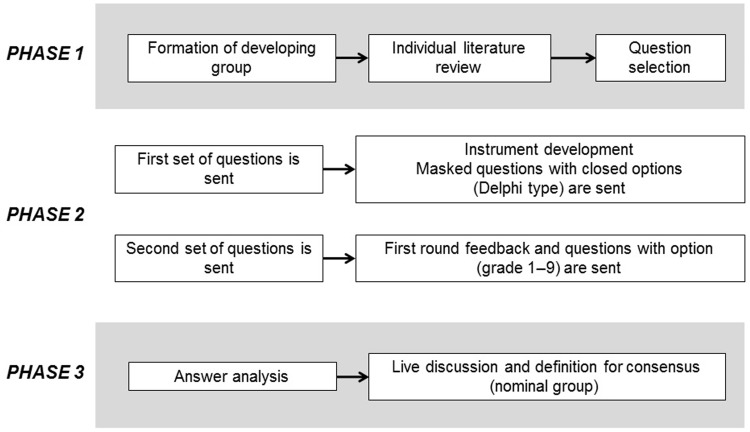
Procedure of expert consensus.

A group of four neurologists, two pulmonologists, two epidemiologists, and two infectious disease specialists was formed. The development of the project was performed in three phases (Figure 1). In Phase 1, the developer group structured the questions which were submitted to experts using Google Forms as a tool for the development of two questionnaires. In Phase 2, questions from Phase 1 were sent via email to the authors, Delphi methodology was used to keep the process blind. Fifteen days later the group of experts was informed of the results in order to provide feedback and to give opportunities to adjust initial responses. The rating system for each option was given on the Likert scale between 1–9 (1: extremely inappropriate, 2 and 3: usually inappropriate, 4, 5, and 6: wrong, 7 and 8: usually appropriate, and 9: extremely appropriate). In this round, a matrix of information was built to analyze the answers by means of median and interquartile ranges (IQRs). The answers scored with median and IQR from 1–3 and 7–9 were considered consensual and the remaining were presented at the nominal group to define consensus. In Phase 3 (nominal), all experts were invited to discuss and argue the nonconsensual answers. At the end of the nominal consensus, the experts provided the reviewed literature for inclusion in this manuscript.

## Results

There were 18 questions for both groups: 13 for pulmonologists and infectious disease specialists with 57 options, and five for neurologists with 27 options, these questions were scored in the second phase (Delphi consensus). Twelve questions remained for the nominal phase for pulmonologists and infectious disease specialists and five for neurologists.

### What drugs for MS require screening for latent TB?

Latent TB screening is suggested for patients with MS who will be started on teriflunomide, fingolimod, natalizumab, alemtuzumab, rituximab, ocrelizumab, daclizumab, or dimethyl fumarate.^15,16^ Screening tests recommended for latent TB are presented in question 3.^17^ Screening tests are not necessary for intramuscular (i.m.) and subcutaneous (s.c.) interferon, interferon β1b or glatiramer acetate, unless there exist other risk factors for conversion of infection to TB disease. No screening is recommended when methyl prednisolone is administered to treat relapses.^8,9,18,19^

### In addition to the drugs included in the previous question, what other conditions should be considered for latent TB screening?

Patients with HIV, subjects in close contact with patients with active TB, silicosis patients, captive populations (prisoners, elderly people, and military personnel), immunocompromised patients due to medical conditions (renal dialysis patients in cancer treatment and uncontrolled diabetes), and patients with a history of addiction to psychoactive drugs. It is also indicated in patients with chronic respiratory diseases and professionals and students of health sciences.^20^

### What screening tests are recommended for diagnosing latent TB in MS patients?

#### Tuberculin skin test (TST)

Also known as purified protein derivative (PPD) or tuberculin test, is suggested as the first choice for the diagnosis of latent TB due to its cost-benefit ratio. This test has a sensitivity of 75% for the diagnosis of infection with *M. tuberculosis* in patients not vaccinated with Bacillus Calmette–Guérin (BCG), 59% in vaccinated patients,^21,22^ and its specificity does not exceed 75%, given the false positives that occur with other mycobacterial infections and vaccination with BCG.^23^ The intradermal test is applied on the forearm and is considered positive when its transverse diameter, measured 72 h later, is ≥10 mm in immunocompetent persons, or ≥5 mm in immunocompromised persons.^20^ It has the disadvantage of a variety of factors that affect the outcome and its interpretation as the standardization of technology, compliance with patient return (set to 72 h), training of the subject who applies the test and who performs reading and history of vaccination (BCG) after first year of life. For reasons related to cost and availability, this tool is the first choice for latent TB diagnosis in our population A negative TST does not rule out latent TB at any age and especially not in immunocompromised host; for this when the risks of infection, of progression to disease and of a poor outcome are high, interferon gamma release tests (IGRAs) may be used as a supplementary diagnostic to enhance the overall sensitivity.

#### IGRA tests

These assays detect the release of interferon gamma in response to specific antigens of *M. tuberculosis.* The most commonly used IGRA tests are QuantiFERON and T-SPOT.TB which have a sensitivity of 76% and 90%, respectively, and a specificity above 95%.^20,24,25^ Although IGRAs have better specificity than the tuberculin test, and are not affected by BCG vaccination under normal conditions, they are not recommended as the first step in the diagnosis of infection due to their higher cost and lower availability.^25^ These assays might be used in situations of high clinical suspicion of latent TB or high risk of progression from infection to disease in presence of negative PPD, which is common in immunocompromised patients.^15,17,24^

**Figure 2.**
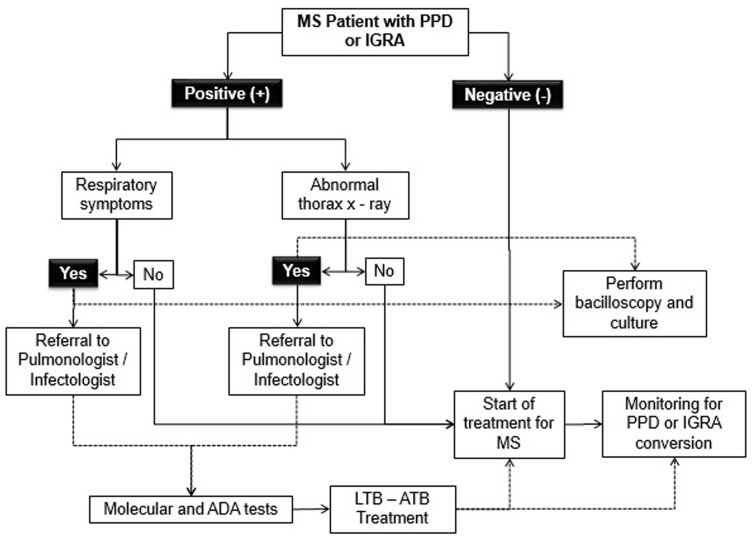
Algorithm for screening and clinical pathway for multiple sclerosis (MS) patient diagnosis and treatment of LTB: latent tuberculosis and ATB: active tuberculosis. IGRA: interferon gamma release test; PPD: purified protein derivative; ADA: adenosine deaminase.

In cases of positive screening, therapeutic interventions for MS should not be delayed until finishing TB treatment, in this setting a comprehensive approach must be offered to control latent TB in the presence of MS. (Figure 2). Repeating an IGRA or performing a TST might be useful when the initial IGRA result is indeterminate, borderline, or invalid and a reason for testing persists.^26^

### What are the treatment schemes suitable for latent TB?

Due to relative efficiency, availability, and costs, administration of isoniazid 300 mg/day is recommended for 9–12 months.^20^ However, any of the regimens recommended by the WHO can be used, including rifampicin 600 mg/day for four months or rifapentine administered 900 mg weekly for a period of three months.^20,27–29^ Although these treatments have similar efficacy and costs, possible adverse events and availability of the tests should be considered. Regimens containing rifampin should be considered for persons who are likely to have been exposed to an isoniazid resistant strain of *M. tuberculosis*.^30^

At this time, limited evidence is available to inform decision-making about the optimal approach to individuals likely to have MDR-TB exposure. WHO recommend strict clinical observation and close monitoring for the development of active TB disease for at least two years is preferred over the provision of preventive treatment for contacts with MDR-TB cases while other authors and Canadian guidelines recommend using fluoroquinolones for nine months in MDR-TB-infected contacts, based upon the drug resistance profile of the presumptive source case.^31,32^ We consider that both recommendations are acceptable.

### How long after LTM treatment initiation should MS drug (DMT) be started?

Considering the potential risk of treatment-related hepatotoxicity with both anti-TB and MS treatments (Table 1), we recommend starting MS DMTs four to eight weeks after treatment initiation for LTB. This period could be modified depending on the risk of hepatotoxicity of the MS treatment. Although both therapies could be started simultaneously, in the case of abnormal hepatic function it would be challenging to differentiate the etiologic factor. According to the above, the panel recommends to start the anti-TB drugs first (Table 1).^15–17,30,33–36^

**Table 1.** Risk of hepatotoxicity of approved drugs for treatment of multiple sclerosis (MS) and latent tuberculosis (TB).

Medication	Hepatotoxicity risk
Interferon B1a 30 mcg, injection once weekly	0–1.9%^31,32^
Interferon B1a 44 mcg, injection three times a week	3.8–11%^33^
Interferon B1b injection every other day	11.3%^34^
Glatiramer acetate (GA)	0%^8^
Natalizumab	5%^8^
Fingolimod	5.5–15.8%^15^
dimethyl fumarate 12	6%^8^
Teriflunomide	14%^16^
Alemtuzumab	2.3%^8^
Isoniazid	0.6% ^36^
Isoniazid plus rifampicin	2.7% ^26^

### How often should clinical and laboratory monitoring be performed in patients with MS who start an anti-TB treatment for latent TB?

Clinical and laboratory monitoring should be performed monthly during the first three months and then every three months until the end of the anti-TB treatment.

If clinical features suggestive of active disease are detected during the follow-up, bacteriological (smear and culture), molecular, and/or histopathologic laboratory tests should be considered. If there are elevated liver function tests it is necessary to assess the existence of factors that increase the risk of hepatotoxicity such as: history of alcoholism, age (35 years and older), obesity, concomitant hepatotoxic drugs, and history of liver disease. If there is at least one of the above conditions besides age, the monthly monitoring must be extended throughout the time of the anti- TB therapy and, in some cases, more frequent monitoring may be required. The chosen drug for MS and its risk of hepatotoxicity (Table 1) along with the factors mentioned above may influence the periodicity of monitoring.^30^

### How often should screening for latent TB be repeated in patients who are treated with an immunosuppressant drug

In patients with initial negative screening who start on an MS drug which can increase risk, latent TB screening should be repeated annually. This annual monitoring allows the attending physician to identify the conversion of tuberculin skin test (PPD) or IGRA. In case of conversion to positive results, anti-TB treatment must be started according to recommendations, to reduce the risk of progression to active TB.^22^

The efficiency of both the PPD and IGRA, is influenced by the immunological condition,^20,21^ that is, sensitivity values increase when the lymphocyte count is greater than 600 mm^3^ and decreases when it is less than 200 mm^3^.^33^

### At what transaminase values is it recommended to modify treatment for latent TB?

Treatment should be modified when transaminase values are greater than three times the normal value and there are hepatic comorbidities such as history of hepatitis, cirrhosis, and alcoholism or concomitant liver symptoms. In the absence of clinical manifestations, treatment should be modified when liver enzymes tests reach five times the normal value. It is necessary, however, to tailor decisions according to every clinical setting.^37^

### What action should be taken when elevated transaminase is identified in the MS patient being treated for latent TB?

When transaminase levels are higher than normal limit (alanine aminotransferase (ALT) more than five times or three times with symptoms such as: abdominal pain, nausea, vomiting, jaundice, or unexplained fatigue), it is recommended to temporarily suspend the drug for latent TB and restart when transaminases are at normal values.^37^ If transaminase values remain elevated, other causes must be assessed, including factors related to the MS treatment. If it is considered that the risk of hepatotoxicity associated with the anti-TB medication exceeds its benefit, the treatment should be stopped. In this case, it is necessary to clearly explain the decision and the potential outcomes to the patient and their family. In addition to liver monitoring other side effects such as skin reactions, polyneuropathies due to pyridoxine deficiencies, cognitive impairment, and lethargy must be considered when isoniazid is prescribed.

### What is the recommended action if active TB is confirmed in a patient receiving immunosuppressant drugs for MS?

When active TB is confirmed in a patient who is being treated for MS with immunosuppressant medications, the MS treatment should be stopped and started again after the first phase of the treatment for the infection is completed.^17^ This decision should be made jointly by the neurologist, pulmonologist, and infectious disease specialist, looking for a balance between the risks and benefits from treatments and the risks associated with the MS and the active TB infection.

This recommendation is based on research which reports 17.5 (IQR: 10–23.5) days to obtain negative results from smear microscopy and 38.5 (IQR 33–43.5) days for sputum culture. However, 10% of these patients take more than 93 days to become negative.^20^

### What treatment scheme is recommended in case of confirmation of active TB?

These patients must be treated according to the National Tuberculosis Program (Colombia). This nine-month scheme includes four drugs in the first eight-week phase: isoniazid, rifampicin, pyrazinamide, and ethambutol, and two drugs in a seven-month phase: isoniazid and rifampicin. In normal conditions, during the second phase the drugs are given three times a week. Under conditions of immunosuppression, especially in the presence of HIV co-infection, daily administration is recommended during the second phase. There is no information on how to manage the second phase in patients with MS. It is recommended for the treatment to be supplied on a daily basis or alternatively three times a week. If the patient has a drug-resistant TB, the use of schemes regulated by the Ministry of Health of Colombia is recommended.^38,39^

### What is the clinical follow-up for MS patients starting treatment for active TB?

It is suggested to perform clinical monitoring with transaminase levels on a monthly basis in the first and second phase of anti-active TB treatment. If hepatotoxicity is identified, treatment should be discontinued until liver function normalizes, to restart later gradually. If this is not possible a treatment with non-hepatotoxic or low-hepatotoxicity medications should be instituted (quinolones, aminoglycosides, ethambutol, amoxicillin-clavulanate, etc.).^39^

## Discussion

The treatment for MS includes a number of therapeutic options.^9^ Due to their effect on the immune system, some of these drugs may be associated with an increased risk of infection including TB.^8,9^ In case of TB, both infection and progression of latent TB to an active disease can occur. For this reason, standard clinical care should include evaluating MS patients for the presence of latent TB and identifying risk factors for conversion to active TB and hepatotoxicity.

Screening for TB infection with the tuberculin skin test (PPD) and, alternatively or additionally with IGRA testing, is mandatory in patients who are immunocompromised and/or who are subjected to immunosuppressive therapy. This is the case of patients with MS who will receive any medications that increase the risk of conversion from latent to active TB.

Confirming this diagnosis in these patients indicates treatment for latent TB once an active disease has been ruled out. Treatment for latent TB significantly reduces the risk of developing active disease and is justified in virtually all cases.

Given the inherent risk of hepatotoxicity in the treatment of latent TB, it is necessary to perform clinical and liver function (transaminase level) monitoring periodically, especially because the MS treatment may also be associated with a risk of hepatotoxicity. The same is true for patients with liver disease or other conditions associated with increased risk for hepatoxicity.

This consensus statement comprises a formal document on what should be considered with regard to diagnosis and management of latent TB in MS patients. Although this information is applicable to the clinical practice, it is possible to identify limitations which could be overtaken when evidence based guidelines are developed for precise diagnostic and treatment strategies for patients with MS and latent TB.

## Declaration of conflicts of interest

The author(s) declared no potential conflicts of interestwith respect to the research, authorship, and/or publicationof this article.
